# Grandmaternal Obesity and Risks of Birth Asphyxia‐Related Complications in Grand‐Offspring: A Countrywide Three‐Generation Study

**DOI:** 10.1002/oby.70046

**Published:** 2025-09-18

**Authors:** Eduardo Villamor, Amanda K. Miglin, Sven Cnattingius

**Affiliations:** ^1^ Department of Epidemiology School of Public Health, University of Michigan Ann Arbor Michigan USA; ^2^ Section of Clinical Epidemiology, Department of Medicine Solna Karolinska Institutet Stockholm Sweden

**Keywords:** Apgar score, BMI, grand‐offspring, grandmother, intergenerational, obesity

## Abstract

**Objective:**

We investigated the associations of grandmaternal early pregnancy BMI with grand‐offspring risks of birth asphyxia‐related complications.

**Methods:**

In a nationwide three‐generation Swedish cohort, we estimated adjusted relative risks (RRs) of Apgar score 0–3 at 5 min and neonatal seizures for categories of grandmaternal BMI among 315,461 maternal and 203,522 paternal singleton live‐born grand‐offspring. To address unmeasured confounding by shared familial factors, we used the parental full sisters' BMI as a negative control exposure. In the maternal line, we assessed whether associations with grandmaternal obesity were mediated through maternal obesity.

**Results:**

Compared with normal maternal grandmaternal BMI, RRs (95% CI) of low Apgar score were, respectively, 1.29 (1.06, 1.57) and 1.53 (1.03, 2.28) for overweight (BMI 25.0 to 29.9) and obesity (BMI ≥ 30.0). For neonatal seizures, the corresponding RRs (95% CI) were 1.32 (1.05, 1.66) and 1.81 (1.17, 2.79). Maternal sisters' BMI was unrelated to both outcomes. Maternal obesity mediated < 25% of the associations with maternal grandmaternal obesity. Paternal grandmaternal obesity was related to an increased risk of neonatal seizures; paternal sisters' BMI was unrelated to this outcome.

**Conclusions:**

Grandmaternal overweight and obesity are related to increased risks of severe birth asphyxia‐related complications in grand‐offspring, independent of unmeasured shared familial factors.

## 1 Introduction

Birth asphyxia, the failure to initiate or sustain breathing at birth, accounts for one in every four neonatal deaths worldwide [1]. Surviving infants are at increased risk of subsequent neurological sequelae resulting from hypoxemic brain damage [2, 3]. Identifying new risk factors for birth asphyxia would enhance the knowledge needed for risk profiling and prevention.

Maternal overweight and obesity have been related to the risk of severe asphyxia‐related complications [4], including a low Apgar score at 5 min after birth [5] and neonatal seizures [6]. The role of maternal obesity on birth asphyxia may be explained through its adverse effects on pregnancy and neonatal outcomes, including increased risks of preeclampsia, preterm birth, large‐for‐gestational‐age size, and traumatic delivery. Notwithstanding, obesity is highly heritable [7]; hence, some of the birth asphyxia risk related to maternal obesity could emanate from obesity in the previous generation. Grandmaternal obesity could be statistically associated with grand‐offspring health outcomes as a result of uncontrolled confounding by factors (genetic or environmental) shared within families or through causal effects possibly due to epigenetic alterations of gene expression that are transmitted across generations [8]. A specific association with grand‐offspring risk of birth asphyxia is plausible because grandmaternal obesity has been related to increased risk of causes and consequences of birth asphyxia, such as preterm birth [9] and neonatal mortality [10], respectively. Nevertheless, the role of grandmaternal obesity on grand‐offspring outcomes related to birth asphyxia has not been previously studied.

Using data from a nationwide three‐generation cohort, we examined the associations of parental grandmaternal (F0 generation) body mass index (BMI) in early pregnancy with grand‐offspring (F2 generation) risks of low Apgar score at 5 min and neonatal seizures, two severe asphyxia‐related outcomes. To address confounding by unmeasured genetic and environmental factors shared within families, we used BMI of the F2 offspring aunts (parental full sisters) as a negative control exposure (NCE).

## 2 Methods

### 2.1 Study Design

We carried out a nationwide multigeneration cohort study of singleton grand‐offspring (F2) who were live‐born between 1983 and 2016, along with their mothers or fathers (F1) and grandmothers (F0) as recorded in the Swedish Medical Birth Register (SMBR), which includes high quality data on > 98% of all births in Sweden [11]. The data sources and assembling of this three‐generation cohort have been described in detail elsewhere [10]. Briefly, grandmothers were identified in the SMBR through individual national registration numbers (NRNs) assigned to all Sweden residents at birth or immigration [12]. There were 3,482,887 singleton births from 1983 to 2016. Exclusions involved invalid NRNs (*n* = 37,193), missing birth dates (*n* = 163), and stillbirths (*n* = 11,691). In the remaining subset, there were 354,888 infants of mothers or fathers whose gestations were registered in the SMBR as pregnancies. The maternal line comprised 315,461 infants (F2 grand‐offspring) of 197,399 mothers (F1) born to 176,766 maternal grandmothers (F0); birth year ranges (median) for F2, F1, and F0 generations were, respectively, 1997–2016 (2013), 1983–2003 (1986), and 1931–1984 (1960). The paternal line consisted of 203,522 infants (F2 grand‐offspring) of 135,452 fathers (F1) born to 124,840 paternal grandmothers (F0); the corresponding generation birth year ranges (median) were 1998–2016 (2014), 1983–2001 (1986), and 1937–1983 (1959). Information on country of birth and educational attainment was obtained through individual record linkages with the Swedish Total Population [13] and Education [14] Registers, respectively.

To implement NCE analyses, we identified the F2 offspring aunts using the Multi‐generation Register [15]. We linked all maternal or paternal (F1) full sisters with ≥ 1 live birth in the SMBR. Among the F1 mothers in the cohort, 59,619 (with 101,053 F2 offspring) had ≥ 1 full sister in the SMBR. Among the F1 fathers, 22,891 (with 35,716 F2 offspring) had ≥ 1 full sister. The F2 aunt's BMI was estimated as the median early pregnancy BMI across all pregnancies from maternal or paternal sisters.

### 2.2 Ethics Approval and Consent to Participate

We sought and obtained approval from the Regional Ethical Review Board in Stockholm, Sweden (No. 2018/5:2). Informed consent was not required because all data were anonymous. All methods were performed in accordance with the relevant guidelines and regulations.

### 2.3 Exposure

At the first antenatal visit (median gestation week 10, IQR: 8, 12 for F0 grandmothers), women reported their height and had weight measured in light clothing. These data were available from 1983 to 1989 and from 1992 onward. For women with ≥ 1 pregnancy in the SMBR, we calculated the median height throughout all pregnancies to reduce error [16]; next, we estimated BMI in kg/m^2^ and used conventional categories (< 18.5, underweight; 18.5–24.9, normal; 25.0–29.9, overweight; ≥ 30.0, obesity) [17].

### 2.4 Outcomes

Apgar scores are assessed by the infant's midwife or physician at 1, 5, and 10 min from birth and recorded in standardized forms that are forwarded to the SMBR after the mother and infant are discharged from the hospital. Diagnoses of neonatal complications are classified according to the Swedish version of the International Classification of Diseases, Tenth Revision (ICD‐10) since 1997. Low Apgar score at 5 min (0–3) in F2 offspring was defined as an Apgar score of 0–3 at both 1 and 5 min to maximize validity. Apgar score at these two time points was available for > 99% in maternal (*n* = 313,833) and paternal (*n* = 202,440) lines. Consensus validity of the Apgar score at 5 min in the SMBR is assessed as “excellent” [11], and predictive validity for neonatal morbidity and mortality is also very high [18, 19, 20]. A comparison of the Apgar score at 5 min between the SMBR and the Swedish Neonatal Quality Register, a separate neonatal register with an action‐oriented ambition for quality improvement, yielded a high agreement (Cohen's kappa = 0.86) [21]. Diagnosis of neonatal seizures was based on ICD‐10 code P90. This code has a positive predictive value of 90% when validated against electroencephalography, amplitude‐integrated electroencephalography, or continuous function monitoring [22].

### 2.5 Covariates

Grandmaternal country of birth was characterized as Nordic (Denmark, Finland, Iceland, Norway, and Sweden) vs. non‐Nordic. Parental age was the delivery date minus the parent's birth date. Grandmaternal or maternal education was the highest completed schooling level. Cohabitation with the father‐to‐be was reported at the first antenatal visit. Parity was the number of births ≥ 22 gestational weeks. Smoking during pregnancy was self‐reported at either the first prenatal visit or in the third trimester; this was validated against cotinine biomarkers in pregnancies occurring between 1996 and 1998, fully pertaining to the F0 generation and partly to the F1 generation [23]. Information on gestational age at birth of the parents (F1) was obtained using the following hierarchy: early second trimester ultrasound (39%), date of the last menstrual period (59%), and a postnatal assessment (2%). Preterm birth was defined as delivery before 37 completed weeks of gestation [24]. The Swedish reference for fetal growth based on ultrasound was used to estimate parental birth weight‐for‐gestational age [25]; this was categorized as < 10th percentile, 10th–90th percentile, or > 90th percentile.

### 2.6 Statistical Analysis

We compared the risk of each outcome according to grandmaternal prepregnancy and parental birth characteristics using relative risks (RRs) with 95% confidence intervals (CI). Next, we estimated RRs of low Apgar scores and neonatal seizures in F2 offspring for early pregnancy BMI levels compared with normal BMI using generalized estimating equations with the Poisson distribution and the log‐link, as well as robust estimates of variance to account for within‐family correlations since the study population included infants from the same family. To estimate the association with grandmaternal (F0) BMI, models were adjusted for grandmaternal year of birth, an important correlate of obesity, and for independent predictors of outcome per preliminary analyses (grandmaternal age at delivery of F1 offspring, education, and smoking). We deliberately excluded postconceptional covariates from the models because their inclusion could lead to overadjustment or collider stratification biases if they lay on the causal path. On the maternal line, we also examined the associations with maternal (F1) early pregnancy BMI, adjusting for maternal grandmaternal early pregnancy BMI and for maternal characteristics previously linked to birth asphyxia such as education level, parity, height, and smoking during pregnancy [4]. On the paternal line, associations were examined with paternal grandmaternal (F0) BMI only because information on adult BMI was unavailable for the fathers. To fully account for potential nonlinearity of the associations, we also considered early pregnancy BMI as a continuous exposure in models that included a linear and three nonlinear terms for BMI as predictors, from restricted cubic splines [26]. Adjusted RRs with 95% CI were estimated for BMI 18.5, 25, and 30, compared with 21.7, the median value for grandmothers.

To address the confounding of the associations between grandmaternal BMI and grand‐offspring birth asphyxia risk by unmeasured shared familial (genetic and environmental) characteristics, we employed the BMI of F2 offspring aunts (i.e., parental full sisters) as a NCE [27], separately in maternal and paternal lines. Assumptions involved a lack of causal effect of the aunt's BMI on the F2 infant's asphyxia risk and the possible presence of a noncausal statistical association through common causes of maternal or paternal (F1) BMI and grand‐offspring (F2) asphyxia, such as grandmaternal (F0) BMI and unmeasured shared familial factors. A lack of association between the parental sisters' BMI and the birth asphyxia of the F2 offspring after adjustment for measured common causes (e.g., grandmaternal BMI) would indicate that uncontrolled shared familial factors are unlikely to play a strong role and strengthen an argument for a causal effect of grandmaternal BMI.

Because early pregnancy BMI was missing in 30% and this could lead to selection bias, we implemented multiple imputation analyses. Ten datasets with predicted missing values were created through a Markov Chain Monte Carlo algorithm which included all available variables (sociodemographic and anthropometric characteristics, pregnancy complications, and delivery information) of the three generations, ignoring their temporal relations [28]. We ran multivariable‐adjusted models on each imputed dataset and combined the estimates per Rubin's rules [29].

In the maternal line, we estimated to what extent the associations of grandmaternal (F0) obesity with grand‐offspring (F2) risk of birth asphyxia‐related outcomes were mediated through maternal (F1) obesity. Lack of mediation could suggest a direct role of intrauterine exposure of the future F1 mother to F0 obesity. We employed causal mediation methods using a counterfactual frame whose assumptions are specified elsewhere [30].

Because associations between grandparental factors and grand‐offspring outcomes may be sex‐specific [31], we conducted supplemental analyses stratified by grand‐offspring's sex. In these analyses, overweight or obesity was considered in a single category (BMI ≥ 25.0) due to limited sample size.

All analyses were conducted with the use of SAS version 9.4 (SAS Institute Inc., Cary, NC).

## 3 Results

### 3.1 Maternal Line

#### 3.1.1 Low Apgar Score at 5 Min

Risk of Apgar score 0–3 at 5 min was 2.0/1000 live births. Maternal grandmaternal (F0) age at daughter's birth < 25 years, education < 12 years, and smoking during pregnancy and maternal (F1) birth weight‐for‐gestational age < 10th percentile were related to increased risk of low Apgar score in grand‐offspring (F2) (Table S1).

Maternal grandmaternal (F0) BMI was positively related to grand‐offspring's risk of low Apgar score at 5 min (Figure S1). Risk/1000 live births increased in a dose–response manner, from 1.7 in the normal grandmaternal BMI category to 2.3 and 2.8 in the overweight and obesity groups, respectively (Table 1). Compared with normal grandmaternal BMI, adjusted RRs (95% CI) after multiple imputation were 1.29 (1.06, 1.57) for overweight and 1.53 (1.03, 2.28) for obesity. Corresponding RRs (95% CI) for maternal (F1) overweight and obesity were, respectively, 1.30 (1.06, 1.58) and 2.18 (1.76, 2.69) (Table 1). Maternal (F1) sisters' BMI was not related to risk of low Apgar score in offspring (F2) in unadjusted or adjusted analyses (Table 1). Twenty percent of the association between maternal grandmaternal obesity and F2 low Apgar risk was mediated through maternal obesity (Table S2). The association did not significantly differ by grand‐offspring sex (Table S3).

**TABLE 1:** Maternal grandmaternal (F0), maternal (F1), and maternal (F1) sisters' early pregnancy BMI and risk of grand‐offspring (F2) Apgar score 0–3 at 5 min after birth in Sweden, 1997–2016.

BMI, kg/m^2^	Number of live births	F2 Apgar score 0–3 at 5 min				
		No.	Risk/1000	Relative risk (95% CI)^a^		
				Unadjusted	Adjusted complete case	Adjusted multiple imputation
Maternal grandmaternal (F0)^b^						
≤ 18.4	17,274	40	2.3	1.36 (0.98, 1.89)	1.36 (0.97, 1.91)	1.17 (0.89, 1.53)
18.5–24.9	163,891	279	1.7	1.00	1.00	1.00
25–29.9	30,756	71	2.3	1.36 (1.05, 1.76)	1.31 (1.00, 1.72)	1.29 (1.06, 1.57)
≥ 30	7165	20	2.8	1.64 (1.04, 2.58)	1.44 (0.89, 2.33)	1.53 (1.03, 2.28)
Missing	94,747	207	2.2			
Maternal (F1)^c^						
≤ 18.4	9094	9	1.0	0.65 (0.34, 1.27)	0.62 (0.28, 1.40)	0.86 (0.47, 1.59)
18.5–24.9	174,561	264	1.5	1.00	1.00	1.00
25–29.9	71,006	136	1.9	1.27 (1.03, 1.56)	1.19 (0.92, 1.54)	1.30 (1.06, 1.58)
≥ 30	40,055	137	3.4	2.26 (1.84, 2.78)	2.10 (1.60, 2.78)	2.18 (1.76, 2.69)
Missing	19,117	71	3.7			
Maternal (F1) sisters^d^						
≤ 18.4	2085	5	2.4	1.15 (0.47, 2.80)	1.34 (0.42, 4.28)	1.43 (0.62, 3.28)
18.5–24.9	58,844	123	2.1	1.00	1.00	1.00
25–29.9	24,760	57	2.3	1.10 (0.81, 1.51)	1.06 (0.69, 1.61)	0.95 (0.69, 1.32)
≥ 30	12,208	22	1.8	0.86 (0.55, 1.36)	0.86 (0.48, 1.54)	0.66 (0.40, 1.07)
Missing	2612	2	0.8			

^a^ From generalized estimating equation models with the Poisson distribution. Grand‐offspring (F2) Apgar score 0–3 at 5 min after birth was the outcome. Robust estimates of variance were specified in all models to account for within‐family correlations.

^b^ Relative risk adjusted for maternal grandmaternal (F0) year of birth, age at daughter's (F1) birth, education level, and smoking during pregnancy. Complete case analyses (*n* = 205,536).

^c^ Relative risk adjusted for maternal grandmaternal (F0) early pregnancy BMI and maternal (F1) education level, parity, height, and smoking during pregnancy. Complete case analyses (*n* = 204,776).

^d^ Relative risk adjusted for maternal grandmaternal (F0) early pregnancy BMI, maternal (F1) early pregnancy BMI, and maternal (F1) height. Complete case analyses (*n* = 65,413).

#### 3.1.2 Neonatal Seizures

Risk of neonatal seizures was 1.9/1000 live births. Maternal grandmaternal birth after 1964 was related to increased risk (Table S4). There was a dose–response association between maternal grandmaternal BMI and risk of neonatal seizures (Figure S2). Risk/1000 live births increased from 1.7 in the maternal grandmaternal normal BMI group to 2.5 and 3.1 in the overweight and obesity categories, respectively (Table 2). In multiple imputation analyses, adjusted RRs (95% CI) for those categories were 1.32 (1.05, 1.66) and 1.81 (1.17, 2.79), respectively. Maternal (F1) overweight and obesity were also significantly related to increased risk of neonatal seizures, but maternal sisters' BMI categories were unrelated to this outcome (Table 2). Twenty‐four percent of the grandmaternal (F0) obesity‐grand‐offspring neonatal seizures association was mediated through maternal (F1) obesity (Table S5). The association was similar for male and female grand‐offspring (Table S6).

**TABLE 2:** Maternal grandmaternal (F0), maternal (F1), and maternal (F1) sisters' early pregnancy BMI and risk of grand‐offspring (F2) neonatal seizures in Sweden, 1997–2016.

BMI, kg/m^2^	Number of live births	F2 neonatal seizures				
		No.	Risk/1000	Relative risk (95% CI)^a^		
				Unadjusted	Adjusted complete case	Adjusted multiple imputation
Maternal grandmaternal (F0)^b^						
≤ 18.4	17,369	31	1.8	1.06 (0.73, 1.54)	1.00 (0.68, 1.49)	1.01 (0.70, 1.44)
18.5–24.9	164,693	277	1.7	1.00	1.00	1.00
25–29.9	30,926	76	2.5	1.46 (1.13, 1.88)	1.41 (1.08, 1.84)	1.32 (1.05, 1.66)
≥ 30	7204	22	3.1	1.82 (1.18, 2.80)	1.64 (1.04, 2.60)	1.81 (1.17, 2.79)
Missing	95,269	181	1.9			
Maternal (F1)^c^						
≤ 18.4	9152	8	0.9	0.57 (0.28, 1.14)	0.52 (0.22, 1.27)	0.67 (0.35, 1.27)
18.5–24.9	175,331	271	1.5	1.00	1.00	1.00
25–29.9	71,383	139	1.9	1.26 (1.03, 1.55)	1.37 (1.08, 1.74)	1.30 (1.06, 1.59)
≥ 30	40,260	127	3.2	2.04 (1.65, 2.52)	1.94 (1.48, 2.54)	2.08 (1.66, 2.60)
Missing	19,335	42	2.2			
Maternal (F1) sisters^d^						
≤ 18.4	2105	5	2.4	1.54 (0.54, 4.44)	1.62 (0.37, 7.14)	1.75 (0.72, 4.27)
18.5–24.9	59,137	91	1.5	1.00	1.00	1.00
25–29.9	24,905	42	1.7	1.10 (0.76, 1.58)	0.87 (0.54, 1.39)	0.91 (0.62, 1.32)
≥ 30	12,270	28	2.3	1.48 (0.97, 2.26)	1.16 (0.66, 2.03)	1.00 (0.63, 1.60)
Missing	2636	4	1.5			

^a^ From generalized estimating equation models with the Poisson distribution. Grand‐offspring (F2) neonatal seizures was the outcome. Robust estimates of variance were specified in all models to account for within‐family correlations.

^b^ Relative risk adjusted for maternal grandmaternal (F0) year of birth, age at daughter's (F1) birth, education level, and smoking during pregnancy. Complete case analyses (*n* = 206,570).

^c^ Relative risk adjusted for maternal grandmaternal (F0) early pregnancy BMI and maternal (F1) education level, parity, height, and smoking during pregnancy. Complete case analyses (*n* = 205,740).

^d^ Relative risk adjusted for maternal grandmaternal (F0) early pregnancy BMI, maternal (F1) early pregnancy BMI, and maternal (F1) height. Complete case analyses (*n* = 65,724).

### 3.2 Paternal Line

Paternal grandmaternal characteristics were not related to low Apgar score at 5 min; paternal birth weight‐for‐gestational age > 90th percentile was related to increased risk (Table S7). Paternal grandmaternal BMI was not associated with risk of grand‐offspring's low Apgar score at 5 min (Table S8).

Paternal grandmaternal non‐Nordic origin was related to lower risk of neonatal seizures (Table S9). Paternal grandmaternal early pregnancy BMI was associated with increased risk of neonatal seizures in a dose–response manner (Table 3, Figure S3). Neonatal seizures risk/1000 live births was 1.6, 2.6, and 3.4 in paternal grandmaternal normal BMI, overweight, and obesity categories, respectively. Compared to normal BMI, adjusted RRs (95% CI) from multiple imputation analyses were, respectively, 1.49 (1.11, 1.99) and 1.95 (1.15, 3.29) for the overweight and obesity groups (Table 3). Paternal (F1) sisters' obesity was unrelated to risk of neonatal seizures. The association between paternal grandmaternal overweight or obesity and grand‐offspring neonatal seizures did not vary significantly by sex of grand‐offspring (Table S10).

**TABLE 3:** Paternal grandmaternal (F0) and paternal (F1) sisters' BMI and risk of grand‐offspring (F2) neonatal seizures in Sweden, 1997–2016.

BMI, kg/m^2^	Number of live births	F2 neonatal seizures				
		No.	Risk/1000	Relative risk (95% CI)^a^		
				Unadjusted	Adjusted complete case	Adjusted multiple imputation
Paternal grandmaternal (F0)^b^						
≤ 18.4	10,945	27	2.5	1.57 (1.05, 2.35)	1.46 (0.95, 2.24)	1.23 (0.84, 1.80)
18.5–24.9	109,404	172	1.6	1.00	1.00	1.00
25–29.9	20,889	54	2.6	1.64 (1.21, 2.23)	1.65 (1.20, 2.27)	1.49 (1.11, 1.99)
≥ 30	4389	15	3.4	2.17 (1.28, 3.68)	2.03 (1.16, 3.57)	1.95 (1.15, 3.29)
Missing	57,895	108	1.9			
Paternal (F1) sisters^c^						
≤ 24.9	20,528	43	2.1	1.00	1.00	1.00
25–29.9	9230	15	1.6	0.78 (0.43, 1.40)	0.78 (0.40, 1.52)	0.73 (0.40, 1.34)
≥ 30	4892	11	2.2	1.07 (0.55, 2.08)	1.04 (0.45, 2.37)	0.97 (0.49, 1.93)
Missing	1066	1	0.9			

^a^ From generalized estimating equation models with the Poisson distribution. Grand‐offspring (F2) neonatal seizures was the outcome. Robust estimates of variance were specified in all models to account for within‐family correlations.

^b^ Relative risk adjusted for paternal grandmaternal (F0) year of birth, age at son's (F1) birth, education level, and smoking during pregnancy. Complete case analyses (*n* = 136,706).

^c^ Relative risk adjusted for paternal grandmaternal (F0) BMI. Complete case analyses (*n* = 24,723).

## 4 Discussion

In a nationwide Swedish cohort comprising three generations, maternal grandmaternal overweight and obesity were related in a dose–response manner to increased risks of two birth asphyxia‐related complications: a low Apgar score at 5 min and neonatal seizures in grand‐offspring. These associations were not explained through confounding by unmeasured factors shared within families. Less than 25% of the associations between grandmaternal (F0) obesity and grand‐offspring (F2) birth asphyxia‐related outcomes were mediated through maternal (F1) obesity. Paternal grandmaternal overweight and obesity were related to increased risk of neonatal seizures, independent of shared familial factors.

We have previously reported that maternal grandmaternal obesity is related to increased risk of neonatal mortality [10]. Because birth asphyxia is an important cause of neonatal death, our current finding suggests one mechanism to explain the intergenerational role of obesity on early mortality. A potential effect of maternal grandmaternal obesity on grand‐offspring risk of birth asphyxia could operate through maternal obesity [4] or its adverse obstetric and perinatal consequences, several of which have been implicated in the etiology of asphyxia‐related neonatal complications [32, 33]. Notwithstanding, maternal obesity explained a relatively small fraction of the association between obesity in the previous generation and birth asphyxia of grand‐offspring. This suggests that other mechanisms may be preponderant.

We may only speculate that one of them pertains to a direct effect of intrauterine exposure of the future F1 mother to obesity in her mother (F0); for example, through epigenetic programming of oocyte gene expression [34, 35]. An effect through maternal obesity, albeit small, could involve intergenerational transmission of obesity and its consequences through intrauterine epigenetic programming of the development of neuroregulatory and metabolic pathways to F1 adiposity [36], and/or postnatal transmission of behaviors [37]. An effect of paternal grandmaternal obesity, if present, would be characterized as transgenerational rather than intergenerational since it cannot be explained through a direct impact on paternal germ cells. It could, however, be explained through alterations in the development of the future father's gonads as a result of early intrauterine exposure to F0 obesity, which may dysregulate spermatogenesis in adulthood [38].

A major line of research on transgenerational and intergenerational influences pertains to the effect of grandparental exposure to famine [39, 40, 41, 42, 43]. Exposure to famine could possibly be connected with underweight in early pregnancy. We did not find any significant associations between grandmaternal underweight and the outcomes of interest; limited statistical power in the BMI < 18.5 category could be one explanation. Another is that the underweight category may not represent women exposed to famine but instead a heterogeneous group including those who are constitutionally thin, persons with wasting from illness, women with eating disorders, etc. Further research on grandmaternal BMI should remain alert to associations with the lower end of the distribution.

There are important strengths to this study. One is its novelty as the first investigation on the role of grandmaternal BMI on the risk of birth asphyxia, a serious, consequential neonatal condition. The use of nationwide population registries reduces selection bias. A prospective design offers many advantages; it avoids reverse causation and precludes differential misclassification of outcome. Objective assessment of body weight reduces information bias from recall. We had the opportunity to adjust for a number of important confounders. Because access to high‐quality health care in Sweden is universal and socioeconomic disparities are less marked than in several other high‐income countries, residual confounding is limited and internal validity is enhanced. The possibility to employ first‐degree relatives as negative control exposures in a nationwide cohort is a very salient advantage.

There are also limitations to note. Inclusion in a cohort spanning three generations by necessity restricts membership to live births, implies survival until reaching reproductive maturity, and requires that the F1 generation be fertile. This set of conditions could lead to survival selection and bias. BMI was missing in ≈30% of grandmothers due to lack of systematic recording of anthropometric data prior to 1992. While findings from multiple imputation analyses were comparable to the main results, selection bias may still be at play. Although analyses were adjusted for grandmaternal year of birth, cohort effects are possible since the prevalence of obesity in Sweden has changed throughout the F1 generation birth years. In addition, while still helpful at the population level, BMI is an imperfect proxy for adiposity and of limited clinical utility as a single indicator. The same BMI value could vary in meaning (e.g., excess adiposity, high muscle mass, pregnancy, edema, etc.) and validity between people, leading to misclassification and to the violation of consistency (single version of treatment), a key assumption for causal inference [44]. This problem may compound cohort effects if the meaning of BMI changed between generations, which is unverifiable. We were unable to consider other birth asphyxia‐related outcomes such as hypoxic–ischemic encephalopathy or meconium aspiration due to limited statistical power. Finally, the generalizability of results to more heterogeneous populations may be limited.

In conclusion, maternal grandmaternal (F0) overweight and obesity in early pregnancy are associated with an increased risk of grand‐offspring's (F2) Apgar score 0–3 at 5 min, whereas both maternal and paternal grandmaternal overweight and obesity are related to an increased risk of neonatal seizures, independent of shared familial factors. The associations of maternal grandmaternal obesity are not substantially mediated through maternal (F1) obesity in early pregnancy, possibly suggesting a direct impact of obesity on the intrauterine development of a future mother's oocytes. These results could contribute to explaining previously reported associations between grandmaternal obesity and neonatal mortality. Future research is warranted on whether grandmaternal obesity effects extend to other consequences of birth asphyxia, such as neurodevelopmental disorders of grand‐offspring.

## Author Contributions

E.V. obtained funding, supervised data analysis, wrote the first draft of the manuscript, and has primary responsibility for the final content. A.K.M. analyzed the data. S.C. provided essential materials including access to the data, contributed to the study design, and aided in the interpretation of findings in the local context. All authors read and approved the final manuscript.

## Conflicts of Interest

The authors declare no conflicts of interest.

## Supporting information


**Data S1:** Supporting Information.

## Data Availability

The data that support the findings of this study are available from the Swedish National Board of Health and Welfare and Statistics Sweden but restrictions apply to the availability of these data, which were used under license for the current study, and so are not publicly available.
