# Supplementary material for: Grandmaternal Obesity and Risks of Birth Asphyxia‐Related Complications in Grand‐Offspring: A Countrywide Three‐Generation Study
Source: Obesity (Silver Spring). 2025 Sep 18;33(12):2398–405. doi: 10.1002/oby.70046 (PMC12636060; doi:10.1002/oby.70046)
Supplement: Supplementary file 1 — Data S1: Supporting Information. [file OBY-33-2398-s001.docx]

Supplementary Table S1. Maternal grandmaternal (F0) and maternal (F1) perinatal characteristics and grandoffspring (F2) Apgar score 0-3 at 5 minutes

|  | Number of live births | F2 Apgar score 0-3 at 5 minutes | | |
| --- | --- | --- | --- | --- |
|  |  | No. | Risk / 1000 | Relative risk  (95% CI)^1^ |
| Overall | 313,833 | 617 | 2.0 |  |
| Maternal grandmaternal (F0) |  |  |  |  |
| Year of birth |  |  |  |  |
| ≤1949 | 16,486 | 35 | 2.1 | 1.04 (0.73, 1.49) |
| 1950–1954 | 43,312 | 69 | 1.6 | 0.78 (0.59, 1.03) |
| 1955–1959 | 86,643 | 147 | 1.7 | 0.83 (0.67, 1.03) |
| 1960–1964 | 98,086 | 200 | 2.0 | 1.00 |
| ≥1965 | 69,306 | 166 | 2.4 | 1.17 (0.96, 1.44) |
| Age at daughter’s (F1) birth, years |  |  |  |  |
| 13–19 | 16,331 | 35 | 2.1 | 1.18 (0.82, 1.70) |
| 20–24 | 98,070 | 221 | 2.3 | 1.24 (1.02, 1.50) |
| 25–29 | 112,148 | 204 | 1.8 | 1.00 |
| 30–34 | 61,238 | 115 | 1.9 | 1.03 (0.82, 1.30) |
| 35–52 | 26,046 | 42 | 1.6 | 0.89 (0.64, 1.23) |
| Country of origin |  |  |  |  |
| Nordic | 295,899 | 584 | 2.0 | 1.00 |
| Non-Nordic | 17,573 | 32 | 1.8 | 0.92 (0.65, 1.32) |
| Missing | 361 | 1 | 2.8 |  |
| Cohabitation with daughter’s father |  |  |  |  |
| Yes | 279,435 | 557 | 2.0 | 1.00 |
| No | 19,140 | 38 | 2.0 | 1.00 (0.72, 1.38) |
| Missing | 15,258 | 22 | 1.4 |  |
| Education, years |  |  |  |  |
| ≤9 | 51,718 | 125 | 2.4 | 1.14 (0.92, 1.40) |
| 10–11 | 131,057 | 279 | 2.1 | 1.00 |
| 12 | 44,993 | 69 | 1.5 | 0.72 (0.55, 0.94) |
| 13–14 | 38,710 | 65 | 1.7 | 0.79 (0.60, 1.03) |
| ≥15 | 46,322 | 76 | 1.6 | 0.77 (0.60, 0.99) |
| Missing | 1033 | 3 | 2.9 |  |
| Parity |  |  |  |  |
| 1 | 124,891 | 235 | 1.9 | 1.00 |
| 2 | 107,385 | 226 | 2.1 | 1.12 (0.93, 1.34) |
| 3 | 54,792 | 94 | 1.7 | 0.91 (0.72, 1.16) |
| ≥4 | 26,765 | 62 | 2.3 | 1.23 (0.93, 1.63) |
| Height, cm |  |  |  |  |
| <155.0 | 8950 | 23 | 2.6 | 1.43 (0.93, 2.22) |
| 155.0–159.9 | 32,184 | 78 | 2.4 | 1.35 (1.03, 1.78) |
| 160.0–164.9 | 83,369 | 171 | 2.1 | 1.14 (0.92, 1.42) |
| 165.0–169.9 | 87,607 | 157 | 1.8 | 1.00 |
| 170.0–174.9 | 57,729 | 105 | 1.8 | 1.01 (0.79, 1.30) |
| ≥175.0 | 18,905 | 31 | 1.6 | 0.92 (0.62, 1.34) |
| Missing | 25,089 | 52 | 2.1 |  |
|  |  |  |  |  |
|  |  |  |  |  |
| Smoking during pregnancy |  |  |  |  |
| No | 193,108 | 356 | 1.8 | 1.00 |
| Yes | 99,781 | 221 | 2.2 | 1.20 (1.02, 1.42) |
| Missing | 20,944 | 40 | 1.9 |  |
| Maternal (F1) perinatal |  |  |  |  |
| Gestational age at birth (weeks) |  |  |  |  |
| Term (≥37) | 298,185 | 583 | 2.0 | 1.00 |
| Preterm (<37) | 15,008 | 33 | 2.2 | 1.12 (0.79, 1.60) |
| Missing | 640 | 1 | 1.6 |  |
| Birthweight-for-gestational age (percentile) |  |  |  |  |
| <10^th^ | 31,129 | 88 | 2.8 | 1.51 (1.20, 1.89) |
| 10^th^ to 90^th^ | 248,764 | 467 | 1.9 | 1.00 |
| >90^th^ | 31,147 | 55 | 1.8 | 0.94 (0.71, 1.24) |
| Missing | 2793 | 7 | 2.4 |  |
|  |  |  |  |  |

Abbreviations: CI, confidence interval.

^1^ From generalized estimating equations with the log link and the Poisson distribution. Robust variances were specified to account for within-family correlations.

Supplementary Table S2. Mediation of the association between maternal grandmaternal (F0) early pregnancy obesity (BMI ≥30) and grandoffspring (F2) risk of Apgar score 0-3 at 5 minutes by maternal (F1) early pregnancy obesity.

| Mediator | Association of maternal grandmaternal (F0) obesity  with Apgar score 0-3 at 5 minutes  Relative risk (95% CI)^1^ | | | %  mediated |
| --- | --- | --- | --- | --- |
|  | Total | Direct | Indirect through mediator |  |
|  |  |  |  |  |
| Maternal early pregnancy obesity (BMI ≥30) | 1.43 (0.88, 2.35) | 1.35 (0.78, 2.34) | 1.06 (0.84, 1.34) | 20 |
|  |  |  |  |  |

Abbreviations: BMI, body mass index; CI, confidence interval.

^1^ Relative risks and 95% CIs are from multivariable-adjusted Poisson regression models. Associations between grandmaternal (F0) obesity and maternal (F1) obesity were modeled with multivariable logistic regression. Covariates included grandmaternal (F0) year of birth, age at delivery of daughter (F1), education level, and smoking during pregnancy. Interaction terms between F0 obesity and mediators were included in all models.

Supplementary Table S3. Maternal grandmaternal (F0) BMI and sex-specific risk of grandoffspring (F2) Apgar score 0-3 at 5 minutes after birth in Sweden, 1997-2016

|  | Number of live births | F2 Apgar score 0-3 at 5 minutes | | | | |
| --- | --- | --- | --- | --- | --- | --- |
|  |  | No. | Risk^1^  / 1000 | Relative risk (95% CI)^1^ | | |
|  |  |  |  | Unadjusted | Adjusted^2^  complete case | Adjusted^2^  multiple imputation |
| Maternal grandmaternal (F0) BMI, kg/m^2^ |  |  |  |  |  |  |
| Male grandoffspring |  |  |  |  |  |  |
| ≤18.4 | 8884 | 27 | 3.0 | 1.53 (1.02, 2.30) | 1.54 (1.02, 2.32) | 1.30 (0.90, 1.87) |
| 18.5-24.9 | 84,334 | 167 | 2.0 | 1.00 | 1.00 | 1.00 |
| ≥25.0 | 19,662 | 46 | 2.3 | 1.18 (0.85, 1.64) | 1.13 (0.81, 1.58) | 1.21 (0.90, 1.62) |
| Missing | 48,781 | 114 | 2.3 |  |  |  |
| Female grandoffspring |  |  |  |  |  |  |
| ≤18.4 | 8391 | 13 | 1.5 | 1.10 (0.62, 1.95) | 1.09 (0.60, 2.00) | 0.99 (0.56, 1.77) |
| 18.5-24.9 | 79,557 | 112 | 1.4 | 1.00 | 1.00 | 1.00 |
| ≥25.0 | 18,259 | 45 | 2.5 | 1.75 (1.24, 2.47) | 1.64 (1.14, 2.36) | 1.47 (1.04, 2.07) |
| Missing | 45,966 | 93 | 2.0 |  |  |  |
|  |  |  |  |  |  |  |
| *P*, interaction |  |  |  | 0.12 | 0.13 | 0.44 |

Abbreviations: BMI, body mass index; CI, confidence interval; RR, relative risk.

^1^ From generalized estimating equation models with the Poisson distribution. Grandoffspring (F2) Apgar score 0-3 at 5 minutes after birth was the outcome. Robust estimates of variance were specified in all models to account for within-family correlations.

^2^ RR adjusted for maternal grandmaternal (F0) year of birth, age at daughter’s (F1) birth, education level, and smoking during pregnancy. Complete case analyses (males n=105,924, females n=99,613).

Supplementary Table S4. Maternal grandmaternal (F0) characteristics and grandoffspring (F2) neonatal seizures

|  | Number of live births | F2 neonatal seizures | | |
| --- | --- | --- | --- | --- |
|  |  | No. | Risk / 1000 | Relative risk  (95% CI)^1^ |
| Overall | 315,461 | 587 | 1.9 |  |
| Maternal grandmaternal (F0) |  |  |  |  |
| Year of birth |  |  |  |  |
| ≤1949 | 16,568 | 31 | 1.9 | 1.10 (0.75, 1.61) |
| 1950–1954 | 43,512 | 76 | 1.7 | 1.03 (0.78, 1.34) |
| 1955–1959 | 87,077 | 160 | 1.8 | 1.08 (0.87, 1.34) |
| 1960–1964 | 98,593 | 168 | 1.7 | 1.00 |
| ≥1965 | 69,711 | 152 | 2.2 | 1.28 (1.03, 1.59) |
| Age at daughter’s (F1) birth, years |  |  |  |  |
| 13–19 | 16,438 | 26 | 1.6 | 0.88 (0.59, 1.33) |
| 20–24 | 98,600 | 199 | 2.0 | 1.13 (0.93, 1.37) |
| 25–29 | 112,710 | 202 | 1.8 | 1.00 |
| 30–34 | 61,539 | 118 | 1.9 | 1.07 (0.85, 1.34) |
| 35–52 | 26,174 | 42 | 1.6 | 0.90 (0.64, 1.25) |
| Country of origin |  |  |  |  |
| Nordic | 297,469 | 558 | 1.9 | 1.00 |
| Non-Nordic | 17,630 | 28 | 1.6 | 0.85 (0.58, 1.24) |
| Missing | 362 | 1 | 2.8 |  |
| Cohabitation with daughter’s father |  |  |  |  |
| Yes | 280,882 | 511 | 1.8 | 1.00 |
| No | 19,233 | 45 | 2.3 | 1.29 (0.95, 1,74) |
| Missing | 15,346 | 31 | 2.0 |  |
| Education, years |  |  |  |  |
| ≤9 | 52,012 | 100 | 1.9 | 0.99 (0.79, 1.25) |
| 10–11 | 131,763 | 255 | 1.9 | 1.00 |
| 12 | 45,212 | 85 | 1.9 | 0.97 (0.76, 1.24) |
| 13–14 | 38,924 | 57 | 1.5 | 0.76 (0.57, 1.01) |
| ≥15 | 46,513 | 86 | 1.8 | 0.96 (0.75, 1.22) |
| Missing | 1037 | 4 | 3.9 |  |
| Parity |  |  |  |  |
| 1 | 125,575 | 243 | 1.9 | 1.00 |
| 2 | 107,893 | 200 | 1.9 | 0.96 (0.79, 1.16) |
| 3 | 55,072 | 88 | 1.6 | 0.83 (0.65, 1.06) |
| ≥4 | 26,921 | 56 | 2.1 | 1.07 (0.80, 1.44) |
| Height, cm |  |  |  |  |
| <155.0 | 8986 | 15 | 1.7 | 0.94 (0.55, 1.59) |
| 155.0–159.9 | 32,368 | 70 | 2.2 | 1.21 (0.92, 1.61) |
| 160.0–164.9 | 83,820 | 164 | 2.0 | 1.10 (0.88, 1.37) |
| 165.0–169.9 | 88,056 | 157 | 1.8 | 1.00 |
| 170.0–174.9 | 58,001 | 110 | 1.9 | 1.06 (0.83, 1.36) |
| ≥175.0 | 19,002 | 25 | 1.3 | 0.74 (0.48, 1.13) |
| Missing | 25,228 | 46 | 1.8 |  |
|  |  |  |  |  |
|  |  |  |  |  |
| Smoking during pregnancy |  |  |  |  |
| No | 194,112 | 354 | 1.8 | 1.00 |
| Yes | 100,283 | 192 | 1.9 | 1.05 (0.88, 1.25) |
| Missing | 21,066 | 41 | 1.9 |  |
| Maternal (F1) perinatal |  |  |  |  |
| Gestational age at birth (weeks) |  |  |  |  |
| Term (≥37) | 299,704 | 553 | 1.8 | 1.00 |
| Preterm (<37) | 15,115 | 33 | 2.2 | 1.18 (0.83, 1.68) |
| Missing | 642 | 1 | 1.6 |  |
| Birthweight-for-gestational age (percentile) |  |  |  |  |
| <10^th^ | 31,306 | 69 | 2.2 | 1.23 (0.96, 1.58) |
| 10^th^ to 90^th^ | 250,064 | 448 | 1.8 | 1.00 |
| >90^th^ | 31,286 | 65 | 2.1 | 1.16 (0.89, 1.50) |
| Missing | 2805 | 5 | 1.9 |  |
|  |  |  |  |  |

Abbreviations: CI, confidence interval.

^1^ From generalized estimating equations with the log link and the Poisson distribution. Robust variances were specified to account for within-family correlations.

Supplementary Table S5. Mediation of the association between maternal grandmaternal (F0) early pregnancy obesity (BMI ≥30) and grandoffspring (F2) risk of neonatal seizures by maternal (F1) early pregnancy obesity.

| Mediator | Association of maternal grandmaternal (F0) obesity  with neonatal seizures  Relative risk (95% CI)^1^ | | | %  mediated |
| --- | --- | --- | --- | --- |
|  | Total | Direct | Indirect through mediator |  |
|  |  |  |  |  |
| Maternal early pregnancy obesity (BMI ≥30) | 1.72 (1.09, 2.71) | 1.54 (0.92, 2.58) | 1.11 (0.88, 1.40) | 24 |
|  |  |  |  |  |

Abbreviations: BMI, body mass index; CI, confidence interval.

^1^ Relative risks and 95% CIs are from multivariable-adjusted Poisson regression models. Associations between grandmaternal (F0) obesity and maternal (F1) obesity were modeled with multivariable logistic regression. Covariates included grandmaternal (F0) year of birth, age at delivery of daughter (F1), education level, and smoking during pregnancy. Interaction terms between F0 obesity and mediators were included in all models.

Supplementary Table S6. Maternal grandmaternal (F0) BMI and sex-specific risk of grandoffspring (F2) neonatal seizures in Sweden, 1997-2016

|  | Number of live births | Neonatal seizures | | | | |
| --- | --- | --- | --- | --- | --- | --- |
|  |  | No. | Risk^1^  / 1000 | Relative risk (95% CI)^1^ | | |
|  |  |  |  | Unadjusted | Adjusted^2^  complete case | Adjusted^2^  multiple imputation |
| Maternal grandmaternal (F0) BMI, kg/m^2^ |  |  |  |  |  |  |
| Male grandoffspring |  |  |  |  |  |  |
| ≤18.4 | 8929 | 15 | 1.7 | 0.87 (0.51, 1.48) | 0.89 (0.52, 1.55) | 0.90 (0.54, 1.50) |
| 18.5-24.9 | 84,747 | 163 | 1.9 | 1.00 | 1.00 | 1.00 |
| ≥25.0 | 19,773 | 56 | 2.8 | 1.47 (1.09, 1.99) | 1.37 (0.99, 1.88) | 1.37 (1.02, 1.83) |
| Missing | 49,034 | 114 | 2.3 |  |  |  |
| Female grandoffspring |  |  |  |  |  |  |
| ≤18.4 | 8440 | 16 | 1.9 | 1.33 (0.79, 2.24) | 1.16 (0.65, 2.05) | 1.15 (0.69, 1.92) |
| 18.5-24.9 | 79,945 | 114 | 1.4 | 1.00 | 1.00 | 1.00 |
| ≥25.0 | 18,357 | 42 | 2.3 | 1.60 (1.13, 2.28) | 1.58 (1.09, 2.28) | 1.42 (1.03, 1.94) |
| Missing | 46,235 | 67 | 1.4 |  |  |  |
|  |  |  |  |  |  |  |
| *P*, interaction |  |  |  | 0.54 | 0.72 | 0.76 |

Abbreviations: BMI, body mass index; CI, confidence interval; RR, relative risk.

^1^ From generalized estimating equation models with the Poisson distribution. Grandoffspring (F2) neonatal seizures was the outcome. Robust estimates of variance were specified in all models to account for within-family correlations.

^2^ RR adjusted for maternal grandmaternal (F0) year of birth, age at daughter’s (F1) birth, education level, and smoking during pregnancy. Complete case analyses (males n= 106,461, females n=100,108).

Supplementary Table S7. Paternal grandmaternal (F0) and paternal (F1) perinatal characteristics and grandoffspring (F2) Apgar score 0-3 at 5 minutes

|  | Number of live births | F2 Apgar score 0-3 at 5 minutes | | |
| --- | --- | --- | --- | --- |
|  |  | No. | Risk / 1000 | Relative risk  (95% CI)^1^ |
| Overall | 202,440 | 388 | 1.9 |  |
| Paternal grandmaternal (F0) |  |  |  |  |
| Year of birth |  |  |  |  |
| ≤1949 | 11,728 | 21 | 1.8 | 0.92 (0.58, 1.46) |
| 1950–1954 | 30,595 | 54 | 1.8 | 0.91 (0.66, 1.25) |
| 1955–1959 | 60,229 | 113 | 1.9 | 0.96 (0.75, 1.24) |
| 1960–1964 | 62,625 | 122 | 1.9 | 1.00 |
| ≥1965 | 37,263 | 78 | 2.1 | 1.07 (0.81, 1.43) |
| Age at son’s (F1) birth, years |  |  |  |  |
| 13–19 | 10,213 | 26 | 2.5 | 1.28 (0.85, 1.94) |
| 20–24 | 60,445 | 111 | 1.8 | 0.92 (0.72, 1.18) |
| 25–29 | 73,471 | 146 | 2.0 | 1.00 |
| 30–34 | 41,100 | 70 | 1.7 | 0.86 (0.64, 1.14) |
| 35–52 | 17,211 | 35 | 2.0 | 1.02 (0.71, 1.48) |
| Country of origin |  |  |  |  |
| Nordic | 191,330 | 371 | 1.9 | 1.00 |
| Non-Nordic | 10,855 | 17 | 1.6 | 0.81 (0.50, 1.31) |
| Missing | 255 | 0 |  |  |
| Cohabitation with daughter’s father |  |  |  |  |
| Yes | 181,869 | 346 | 1.9 | 1.00 |
| No | 11,621 | 31 | 2.7 | 1.40 (0.97, 2.02) |
| Missing | 8950 | 11 | 1.2 |  |
| Education, years |  |  |  |  |
| ≤9 | 32,354 | 60 | 1.9 | 0.89 (0.67, 1.20) |
| 10–11 | 83,953 | 174 | 2.1 | 1.00 |
| 12 | 28,230 | 54 | 1.9 | 0.92 (0.68, 1.25) |
| 13–14 | 25,726 | 39 | 1.5 | 0.73 (0.52, 1.03) |
| ≥15 | 31,477 | 59 | 1.9 | 0.90 (0.67, 1.21) |
| Missing | 700 | 2 | 2.9 |  |
| Parity |  |  |  |  |
| 1 | 80,131 | 176 | 2.2 | 1.00 |
| 2 | 70,128 | 122 | 1.7 | 0.79 (0.63, 1.00) |
| 3 | 35,808 | 59 | 1.6 | 0.75 (0.56, 1.01) |
| ≥4 | 16,373 | 31 | 1.9 | 0.86 (0.59, 1.26) |
| Height, cm |  |  |  |  |
| <155.0 | 5568 | 13 | 2.3 | 1.30 (0.73, 2.30) |
| 155.0–159.9 | 19,745 | 42 | 2.1 | 1.18 (0.83, 1.69) |
| 160.0–164.9 | 51,711 | 102 | 2.0 | 1.09 (0.83, 1.44) |
| 165.0–169.9 | 57,156 | 103 | 1.8 | 1.00 |
| 170.0–174.9 | 38,866 | 83 | 2.1 | 1.19 (0.89, 1.58) |
| ≥175.0 | 12,436 | 20 | 1.6 | 0.89 (0.55, 1.44) |
| Missing | 16,958 | 25 | 1.5 |  |
|  |  |  |  |  |
|  |  |  |  |  |
| Smoking during pregnancy |  |  |  |  |
| No | 125,748 | 241 | 1.9 | 1.00 |
| Yes | 63,520 | 126 | 2.0 | 1.04 (0.83, 1.28) |
| Missing | 13,172 | 21 | 1.6 |  |
| Paternal (F1) perinatal |  |  |  |  |
| Gestational age at birth (weeks) |  |  |  |  |
| Term (≥37) | 191,107 | 372 | 1.9 | 1.00 |
| Preterm (<37) | 10,979 | 16 | 1.5 | 0.75 (0.45, 1.23) |
| Missing | 354 | 0 |  |  |
| Birthweight-for-gestational age (percentile) |  |  |  |  |
| <10^th^ | 14,919 | 32 | 2.1 | 1.18 (0.82, 1.70) |
| 10^th^ to 90^th^ | 161,636 | 293 | 1.8 | 1.00 |
| >90^th^ | 24,147 | 60 | 2.5 | 1.37 (1.04, 1.81) |
| Missing | 1738 | 3 | 1.7 |  |
|  |  |  |  |  |

Abbreviations: CI, confidence interval.

^1^ From generalized estimating equations with the log link and the Poisson distribution. Robust variances were specified to account for within-family correlations.

Supplementary Table S8. Paternal grandmaternal (F0) and paternal (F1) sisters’ BMI and risk of grandoffspring (F2) Apgar score 0-3 at 5 minutes after birth in Sweden, 1997-2016

| BMI, kg/m^2^ | Number of live births | F2 Apgar score 0-3 at 5 minutes | | | | |
| --- | --- | --- | --- | --- | --- | --- |
|  |  | No. | Risk^1^  / 1000 | Relative risk (95% CI)^1^ | | |
|  |  |  |  | Unadjusted | Adjusted  complete case | Adjusted  multiple imputation |
| Paternal grandmaternal (F0)^2^ |  |  |  |  |  |  |
| ≤18.4 | 10,885 | 29 | 2.7 | 1.45 (0.98, 2.14) | 1.46 (0.98, 2.17) | 1.22 (0.85, 1.76) |
| 18.5-24.9 | 108,812 | 200 | 1.8 | 1.00 | 1.00 | 1.00 |
| 25-29.9 | 20,782 | 46 | 2.2 | 1.20 (0.87, 1.66) | 1.19 (0.86, 1.65) | 1.17 (0.86, 1.59) |
| ≥30 | 4363 | 6 | 1.4 | 0.75 (0.33, 1.68) | 0.63 (0.26, 1.53) | 0.85 (0.40, 1.78) |
| Missing | 57,598 | 107 | 1.9 |  |  |  |
| Paternal (F1) sisters^3^ |  |  |  |  |  |  |
| ≤24.9 | 20,414 | 31 | 1.5 | 1.00 | 1.00 | 1.00 |
| 25-29.9 | 9170 | 21 | 2.3 | 1.51 (0.87, 2.62) | 1.63 (0.84, 3.14) | 1.53 (0.87, 2.68) |
| ≥30 | 4863 | 10 | 2.1 | 1.35 (0.66, 2.76) | 1.04 (0.39, 2.78) | 1.42 (0.68, 2.96) |
| Missing | 1057 | 0 |  |  |  |  |

Abbreviations: BMI, body mass index; CI, confidence interval; RR, relative risk.

^1^ From generalized estimating equation models with the Poisson distribution. Grandoffspring (F2) Apgar score 0-3 at 5 minutes after birth was the outcome. Robust estimates of variance were specified in all models to account for within-family correlations.

^2^ RR adjusted for paternal grandmaternal (F0) year of birth, age at son’s (F1) birth, education level, and smoking during pregnancy. Complete case analyses (n=135,968).

^3^ RR adjusted for paternal grandmaternal (F0) BMI. Complete case analyses (n=24,578).

Supplementary Table S9. Paternal grandmaternal (F0) characteristics and grandoffspring (F2) neonatal seizures

|  | Number of live births | F2 neonatal seizures | | |
| --- | --- | --- | --- | --- |
|  |  | No. | Risk / 1000 | Relative risk  (95% CI)^1^ |
| Overall | 203,522 | 376 | 1.8 |  |
| Paternal grandmaternal (F0) |  |  |  |  |
| Year of birth |  |  |  |  |
| ≤1949 | 11,776 | 27 | 2.3 | 1.24 (0.82, 1.89) |
| 1950–1954 | 30,739 | 55 | 1.8 | 0.97 (0.70, 1.34) |
| 1955–1959 | 60,529 | 99 | 1.6 | 0.89 (0.68, 1.16) |
| 1960–1964 | 62,973 | 116 | 1.8 | 1.00 |
| ≥1965 | 37,505 | 79 | 2.1 | 1.14 (0.86, 1.52) |
| Age at son’s (F1) birth, years |  |  |  |  |
| 13–19 | 10,294 | 20 | 1.9 | 1.03 (0.65, 1.65) |
| 20–24 | 60,788 | 113 | 1.9 | 0.99 (0.77, 1.27) |
| 25–29 | 73,845 | 139 | 1.9 | 1.00 |
| 30–34 | 41,302 | 67 | 1.6 | 0.86 (0.64, 1.15) |
| 35–52 | 17,293 | 37 | 2.1 | 1.14 (0.79, 1.63) |
| Country of origin |  |  |  |  |
| Nordic | 192,379 | 366 | 1.9 | 1.00 |
| Non-Nordic | 10,888 | 10 | 0.9 | 0.48 (0.26, 0.90) |
| Missing | 255 | 0 |  |  |
| Cohabitation with daughter’s father |  |  |  |  |
| Yes | 182,824 | 329 | 1.8 | 1.00 |
| No | 11,698 | 26 | 2.2 | 1.24 (0.83, 1.84) |
| Missing | 9000 | 21 | 2.3 |  |
| Education, years |  |  |  |  |
| ≤9 | 32,538 | 52 | 1.6 | 0.78 (0.58, 1.07) |
| 10–11 | 84,439 | 172 | 2.0 | 1.00 |
| 12 | 28,369 | 49 | 1.7 | 0.85 (0.62, 1.16) |
| 13–14 | 25,843 | 43 | 1.7 | 0.82 (0.58, 1.15) |
| ≥15 | 31,629 | 59 | 1.9 | 0.92 (0.68, 1.23) |
| Missing | 704 | 1 | 1.4 |  |
| Parity |  |  |  |  |
| 1 | 80,566 | 157 | 1.9 | 1.00 |
| 2 | 70,478 | 128 | 1.8 | 0.93 (0.74, 1.18) |
| 3 | 36,002 | 66 | 1.8 | 0.94 (0.71, 1.25) |
| ≥4 | 16,476 | 25 | 1.5 | 0.78 (0.51, 1.19) |
| Height, cm |  |  |  |  |
| <155.0 | 5597 | 12 | 2.1 | 1.05 (0.58, 1.91) |
| 155.0–159.9 | 19,867 | 28 | 1.4 | 0.69 (0.46, 1.05) |
| 160.0–164.9 | 51,974 | 107 | 2.1 | 1.01 (0.78, 1.31) |
| 165.0–169.9 | 57,494 | 117 | 2.0 | 1.00 |
| 170.0–174.9 | 39,069 | 69 | 1.8 | 0.87 (0.64, 1.17) |
| ≥175.0 | 12,495 | 17 | 1.4 | 0.67 (0.39, 1.14) |
| Missing | 17,026 | 26 | 1.5 |  |
|  |  |  |  |  |
|  |  |  |  |  |
| Smoking during pregnancy |  |  |  |  |
| No | 126,383 | 233 | 1.8 | 1.00 |
| Yes | 63,896 | 121 | 1.9 | 1.03 (0.82, 1.28) |
| Missing | 13,243 | 22 | 1.7 |  |
| Paternal (F1) perinatal |  |  |  |  |
| Gestational age at birth (weeks) |  |  |  |  |
| Term (≥37) | 192,119 | 362 | 1.9 | 1.00 |
| Preterm (<37) | 11,048 | 14 | 1.3 | 0.67 (0.39, 1.15) |
| Missing | 355 | 0 |  |  |
| Birthweight-for-gestational age (percentile) |  |  |  |  |
| <10^th^ | 15,009 | 30 | 2.0 | 1.09 (0.75, 1.58) |
| 10^th^ to 90^th^ | 162,492 | 299 | 1.8 | 1.00 |
| >90^th^ | 24,275 | 42 | 1.7 | 0.94 (0.68, 1.30) |
| Missing | 1746 | 5 | 2.9 |  |
|  |  |  |  |  |

Abbreviations: CI, confidence interval.

^1^ From generalized estimating equations with the log link and the Poisson distribution. Robust variances were specified to account for within-family correlations.

Supplementary Table S10. Paternal grandmaternal (F0) BMI and sex-specific risk of grandoffspring (F2) neonatal seizures in Sweden, 1997-2016

|  | Number of live births | Neonatal seizures | | | | |
| --- | --- | --- | --- | --- | --- | --- |
|  |  | No. | Risk^1^  / 1000 | Relative risk (95% CI)^1^ | | |
|  |  |  |  | Unadjusted | Adjusted^2^  complete case | Adjusted^2^  multiple imputation |
| Paternal grandmaternal (F0) BMI, kg/m^2^ |  |  |  |  |  |  |
| Male grandoffspring |  |  |  |  |  |  |
| ≤18.4 | 5629 | 14 | 2.5 | 1.33 (0.76, 2.32) | 1.40 (0.80, 2.45) | 1.08 (0.65, 1.81) |
| 18.5-24.9 | 56,606 | 106 | 1.9 | 1.00 | 1.00 | 1.00 |
| ≥25.0 | 12,978 | 41 | 3.2 | 1.69 (1.18, 2.42) | 1.66 (1.13, 2.43) | 1.49 (1.07, 2.07) |
| Missing | 29,955 | 69 | 2.3 |  |  |  |
| Female grandoffspring |  |  |  |  |  |  |
| ≤18.4 | 5316 | 13 | 2.4 | 1.96 (1.08, 3.54) | 1.55 (0.79, 3.05) | 1.47 (0.83, 2.62) |
| 18.5-24.9 | 52,798 | 66 | 1.3 | 1.00 | 1.00 | 1.00 |
| ≥25.0 | 12,300 | 28 | 2.3 | 1.82 (1.17, 2.83) | 1.82 (1.16, 2.84) | 1.65 (1.04, 2.62) |
| Missing | 27,940 | 39 | 1.4 |  |  |  |
|  |  |  |  |  |  |  |
| *P*, interaction |  |  |  | 0.66 | 0.93 | 0.72 |

Abbreviations: BMI, body mass index; CI, confidence interval; RR, relative risk.

^1^ From generalized estimating equation models with the Poisson distribution. Grandoffspring (F2) neonatal seizures was the outcome. Robust estimates of variance were specified in all models to account for within-family correlations.

^2^ RR adjusted for paternal grandmaternal (F0) year of birth, age at daughter’s (F1) birth, education level, and smoking during pregnancy. Complete case analyses (males n=70,628, females n=66,078).

Supplementary Figure S1. Relative risks of grandoffspring (F2) Apgar score 0-3 at 5 minutes according to maternal grandmaternal (F0) early pregnancy body mass index (BMI) in Sweden, 1997-2016.

| **A**  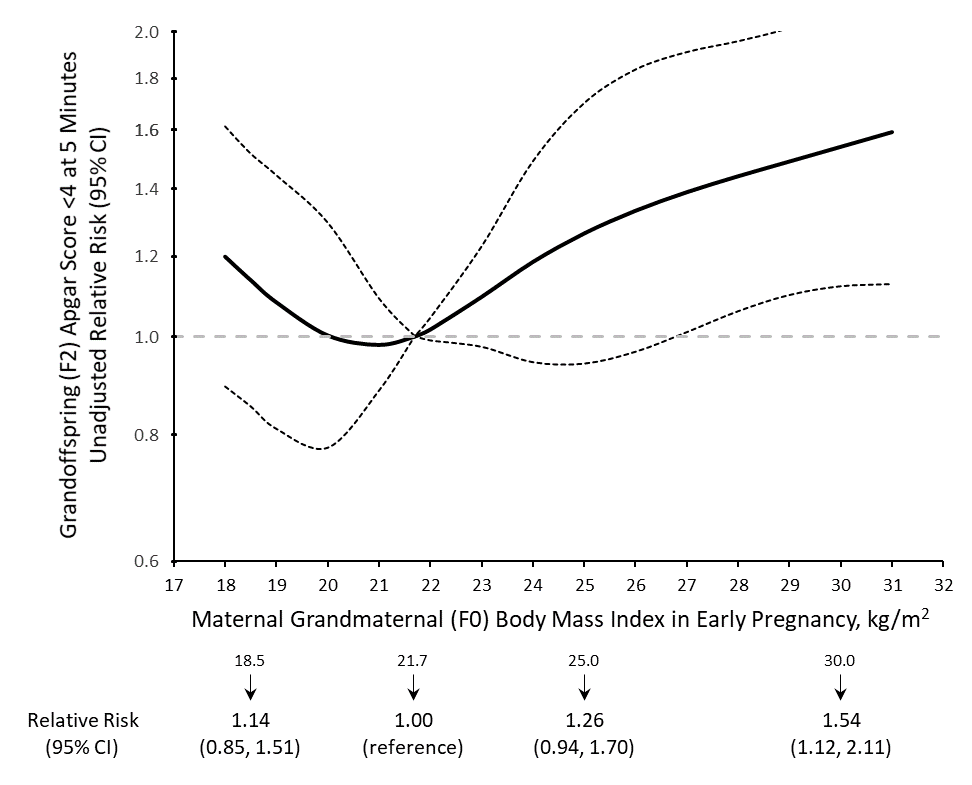 | **B**  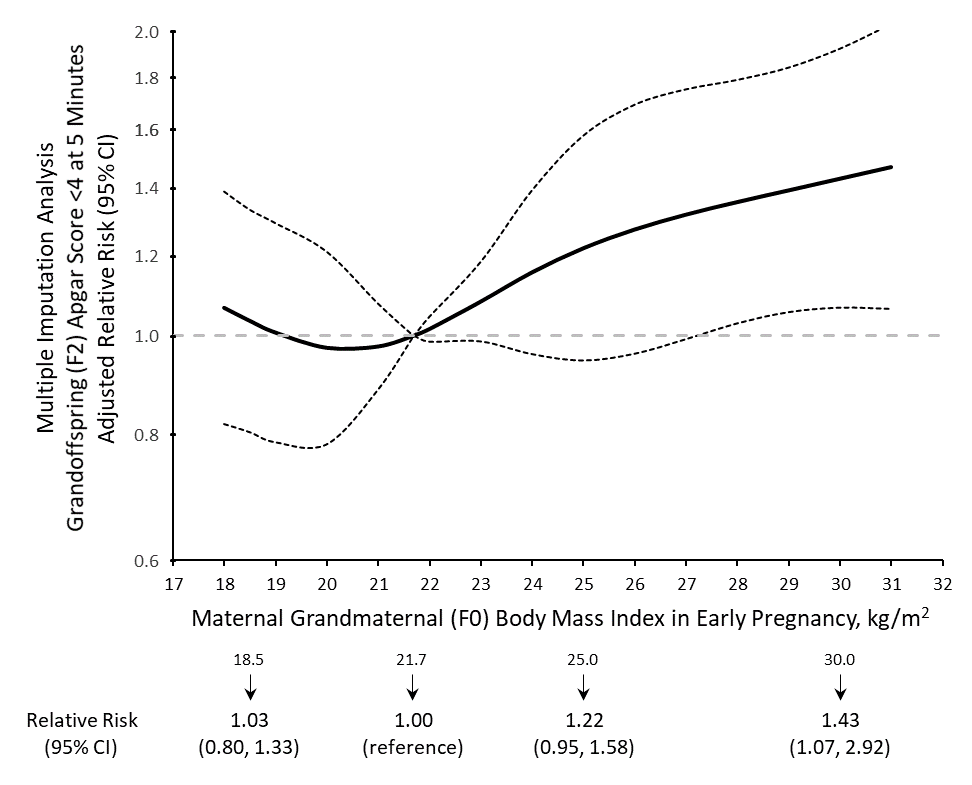 |
| --- | --- |

Supplementary Figure S2. Relative risks of grandoffspring (F2) seizures according to maternal grandmaternal (F0) early pregnancy body mass index (BMI) in Sweden, 1997-2016.

| **A**  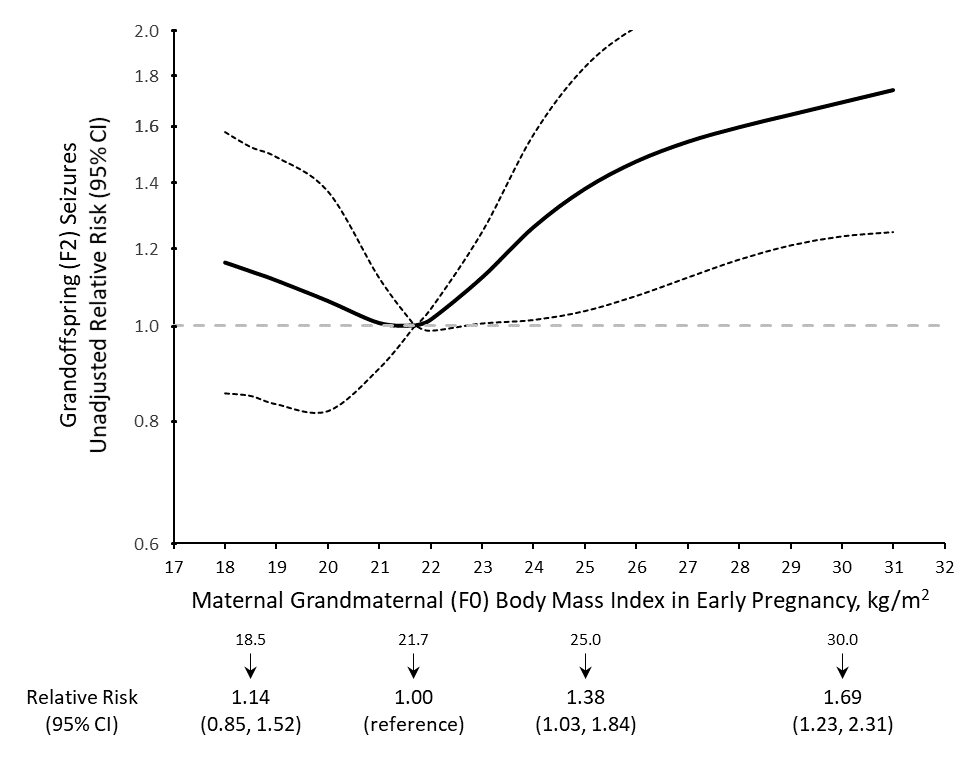 | **B**  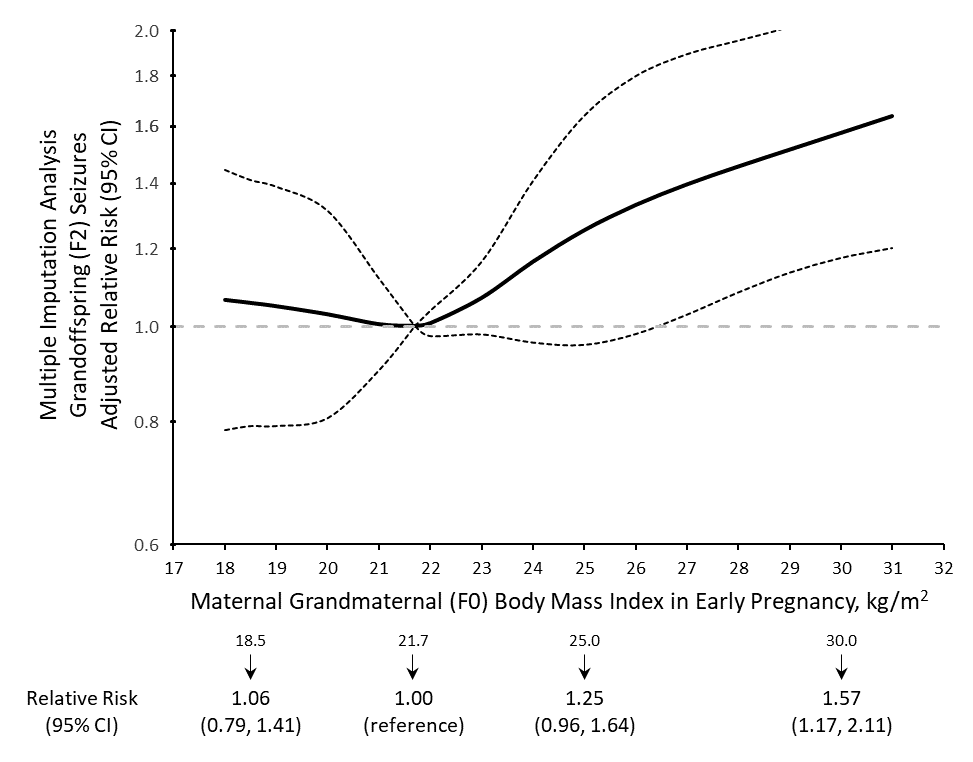 |
| --- | --- |

Supplementary Figure S3. Relative risks of grandoffspring (F2) seizures according to paternal grandmaternal (F0) early pregnancy body mass index (BMI) in Sweden, 1997-2016.

| **A**  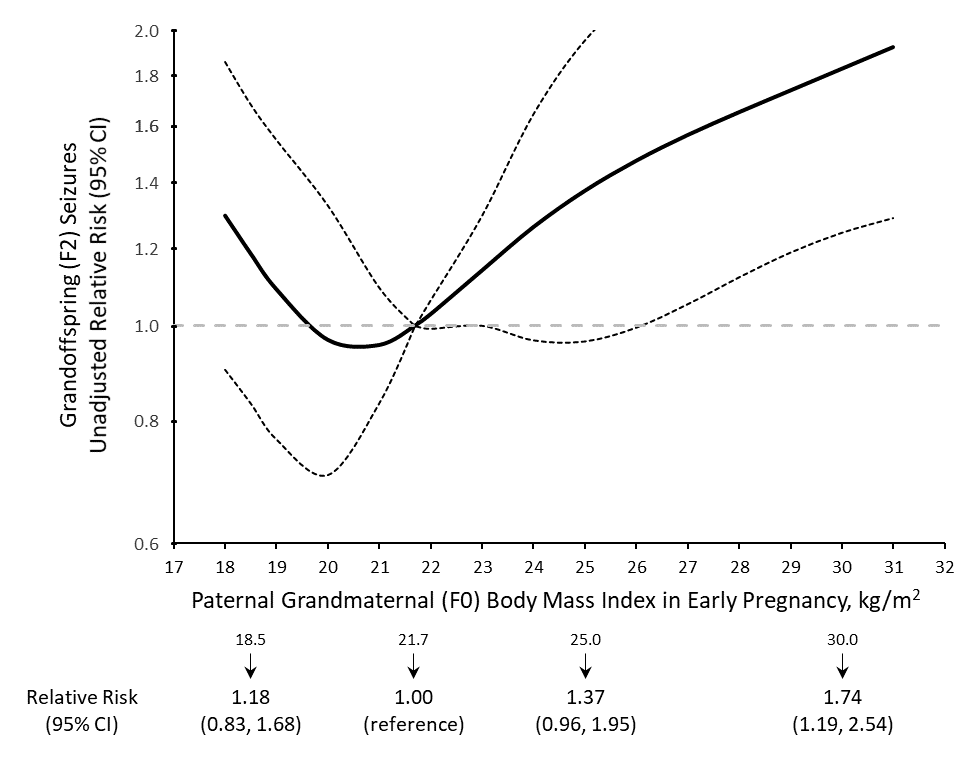 | **B**  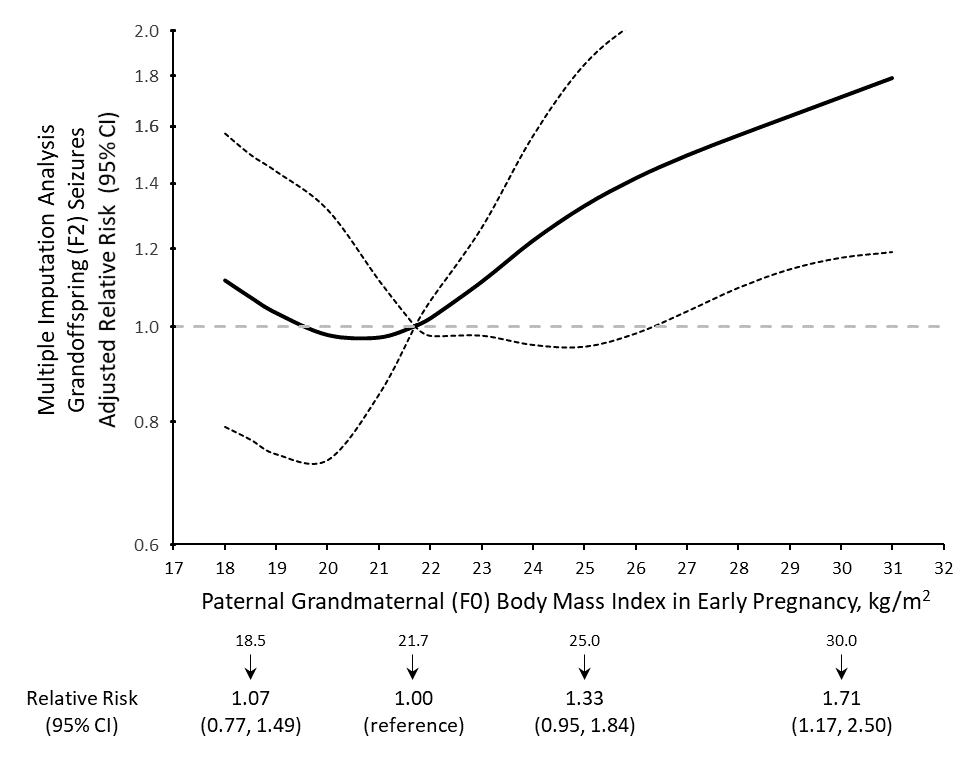 |
| --- | --- |
